# Thiol/Disulfide Homeostasis in Lung Cancer: Insights from a Clinical Study

**DOI:** 10.3390/antiox15010114

**Published:** 2026-01-15

**Authors:** Selen Karaoğlanoğlu, Müge Sönmez, Hüseyin Erdal

**Affiliations:** 1 Department of Pulmonology, Faculty of Medicine, Ordu University, 52200 Ordu, Türkiye; 2 Department of Internal Medicine, Division of Oncology, Faculty of Medicine, Ordu University, Ordu State Hospital, 52200 Ordu, Türkiye; mugesonmez@odu.edu.tr; 3 Department of Medical Genetic, Faculty of Medicine, Aksaray University, 68100 Aksaray, Türkiye; huseyinerdal@aksaray.edu.tr

**Keywords:** oxidative stress, lung cancer, thiol–disulfide homeostasis, oxidants

## Abstract

**Background**: The development of lung cancer is strongly influenced by oxidative stress (OS), which results when the balance between oxidants and antioxidants is disturbed. Evaluation of both specific redox markers such as thiol/disulfide homeostasis (TDH) and overall indicators including total antioxidant status (TAS), total oxidant status (TOS), and oxidative stress index (OSI) may provide a more comprehensive view of oxidative imbalance in lung cancer. We examined OS indices and TDH in patients with lung cancer versus healthy controls. **Methods**: Eighty participants were enrolled, consisting of 40 patients with newly diagnosed lung cancer and 40 age- and sex-matched healthy controls. Serum levels of native thiol (NT), total thiol (TT), and disulfide were determined using an automated spectrophotometric method. Additionally, TAS, TOS, and the OSI were evaluated to provide an overall assessment of oxidative balance. Routine hematological and biochemical parameters were compared between groups. **Results**: White blood cell and neutrophil counts were notably higher in lung cancer patients compared with controls (*p* < 0.05). NT and TT levels were remarkably decreased, whereas disulfide levels, TOS, and OSI were significantly elevated in the lung cancer group (*p* < 0.05). TAS levels tended to be lower in patients, although not reaching statistical significance. No significant association was observed between oxidative parameters and tumor stage or localization. **Conclusions:** Patients with lung cancer exhibited a marked oxidative imbalance, characterized by elevated oxidant burden and impaired TDH. Combined assessment of TAS, TOS, OSI, and thiol/disulfide parameters may provide valuable insight into the oxidative pathophysiology of lung cancer and hold potential as complementary biomarkers for disease evaluation. Further large scale studies are needed to confirm these findings.

## 1. Introduction

Lung cancer is a complex and multifactorial disease, with tobacco smoking, environmental pollutants, genetic predisposition, and chronic inflammation among the major risk factors contributing to its development [1,2]. Over the past decade, significant changes have occurred in the epidemiology and prevention of lung cancer, driven by shifts in smoking habits, groundbreaking discoveries in lung cancer genetics, advances in understanding the role of the immune system in tumor control, and major progress in therapeutic options. Despite these developments, lung cancer remains the leading cause of cancer-related deaths worldwide [3]. Patients are frequently diagnosed at a late stage, which largely accounts for its fatality [4]. Among the biological processes driving the onset and advancement of lung cancer, oxidative stress (OS) stands out as a central mechanism [5]. OS emerges when redox homeostasis shifts toward oxidation, leading to excessive reactive species and inadequate antioxidant defenses. Sustained oxidative imbalance promotes DNA damage, genomic instability, and dysregulation of cellular signaling cascades, thereby facilitating malignant transformation and tumor progression [6]. Therefore, assessing OS parameters and redox balance, such as thiol/disulfide homeostasis (TDH), total antioxidant status (TAS), total oxidant status (TOS), and the oxidative stress index (OSI), may contribute to a deeper comprehension of biological behavior and clinical course of lung cancer. An altered equilibrium between reactive oxygen species (ROS) formation and the effectiveness of mechanisms of antioxidant protection underlies OS, which has been recognized as a pivotal contributor to the pathogenesis of lung cancer and other respiratory disorders [7,8,9]. Elevated ROS levels can induce DNA damage, promote gene mutations, activate oncogenic signaling pathways, and disrupt normal apoptotic mechanisms, thereby facilitating malignant transformation and tumor progression [10].

Homeostasis of thiol and disulfide molecules is essential for the regulation of oxidative and reductive processes within cells and reflects the dynamic balance between antioxidant thiol groups and their oxidized disulfide forms [11,12]. These markers have been increasingly investigated in various malignancies as potential biomarkers for disease presence, progression, and prognosis [6,13,14].

Despite growing evidence supporting the role of OS in lung cancer, most previous studies have evaluated individual redox parameters in isolation, and data integrating dynamic TDH with global OS indices such as TAS, TOS, and OSI remain limited. Moreover, the extent to which these markers jointly reflect systemic redox imbalance in lung cancer has not been fully elucidated. Therefore, the present study aims to provide an integrated assessment of TDH together with TAS, TOS, and OSI in patients with lung cancer compared with healthy controls. By simultaneously evaluating these complementary redox parameters, we sought to achieve a more comprehensive characterization of oxidative imbalance and to explore their potential clinical relevance as supportive biomarkers that may accompany established diagnostic and screening approaches, rather than serve as standalone tools. The findings of this study are intended to be interpreted within an exploratory framework, contributing to improved phenotyping of redox status in lung cancer and generating hypotheses for future, larger-scale investigations.

## 2. Materials and Methods

### 2.1. Study Design

In this prospective observational design, 80 patients participated in the study (40 lung cancer and 40 control groups) in the medical oncology outpatient clinic of Ordu State Hospital between July 2023 and January 2024. Approval for the study was granted by the institutional ethics committee, and the research adhered to the ethical guidelines of the Declaration of Helsinki. Written informed consent was obtained from every participant before enrollment.

Information such as patients’ age, gender, smoking status, cancer subtypes, genetic analyses, presence of metastasis, and location of metastasis was obtained from patient records and the hospital’s automation system.

### 2.2. Inclusion and Exclusion Criterias

Patients were eligible for inclusion if they had a newly and pathologically confirmed diagnosis of lung cancer established by a medical oncologist and had not yet received any chemotherapy. All participants provided written informed consent prior to enrollment.

Patients were excluded if they were younger than 18 years of age, declined to participate, lacked the required hematological data in the hospital database, showed clinical signs of acute infection, had a history of any other malignancy, had previously received anticancer therapy, or presented with chronic inflammatory, hematological, or autoimmune disorders. Additional exclusion criteria included the use of anti-inflammatory agents, corticosteroids, or immunosuppressive drugs within the preceding six months, the use of any supplements that could influence the antioxidant system, and evidence of hepatic or renal dysfunction.

### 2.3. Assessment of Dynamic Thiol–Disulfide Levels

An automated spectrophotometric technique, as previously reported, was employed to determine native thiol (NT) and total thiol (TT) levels [15]. In brief, thiol groups were first reduced with sodium borohydride. To avoid interference with 2,2-dithiobisnitrobenzoic acid (DTNB), surplus sodium borohydride was neutralized and removed from the reaction mixture using formaldehyde. Disulfide concentrations were then determined by subtracting NT levels from TT levels and dividing the difference by two.

### 2.4. Measurements of TAS, TOS, and OSI Levels

TOS and TAS were assessed spectrophotometrically using Erel’s method [16]. The results were described as mmol Trolox equivalent per liter for TAS and mmol H_2_O_2_ equivalent per liter for TOS. Subsequently, the OSI was calculated as follows: OSI (arbitrary unit) = [TOS (mmol H_2_O_2_ Eq/L)/TAS (mmol Trolox Eq/L)] × 100.

### 2.5. Data Collection

Venous blood samples were collected from all participants after an overnight fasting period of at least 8–10 h to minimize the potential influence of recent dietary intake on OS parameters. Patients’ hemogram, C-reactive protein (CRP), urea, creatinine, alanine aminotransferase (ALT), aspartate aminotransferase (AST), lactate dehydrogenase (LDH), NT, TT, and disulfide levels were assessed. For hematological parameters including hemoglobin, white blood cell (WBC), neutrophils, lymphocytes, platelets, monocytes, eosinophils, and mean platelet volume (MPV), approximately 2 mL of blood was drawn from the antecubital vein into EDTA-K2 tubes. Tubes were gently inverted and analyzed with a Sysmex XN 1000 automated hematology analyzer (Sysmex Corporation, Kobe, Japan). For biochemical measurements (CRP, urea, creatinine, ALT, AST, LDH), around 8 mL of blood was collected into tubes without anticoagulant. Samples were centrifuged at 3000 rpm for 10 min and analyzed using a cobas 8000 c 702 automated biochemistry analyzer (Roche Diagnostics, Mannheim, Germany). Serum aliquots were then stored at −80 °C in Eppendorf tubes until NT and TT analyses were performed. Serum TAS, TOS, and OSI levels, as well as TDH parameters (NT, TT, and disulfide levels), were measured using commercially available, automated spectrophotometric methods according to the manufacturer’s instructions. All analyses were performed in the same laboratory under standardized conditions, and laboratory personnel were blinded to the clinical status of the participants.

### 2.6. Statistical Analysis

Data were analyzed statistically using the MedCalc statistical software package (version 20.009; Ostend, Belgium). The Shapiro–Wilk test was applied to evaluate whether the data followed a normal distribution. Categorical variables were presented as frequencies and percentages. Continuous data were expressed as mean, standard deviation (SD), median, 25th percentile, and 75th percentile. For the comparison of laboratory parameters and OS parameters between groups, the Independent t-test was used for groups that followed a normal distribution, and the Mann–Whitney U test was used for groups that did not follow a normal distribution. Data with a normal distribution are shown as mean and SD in the tables, while data that do not follow a normal distribution are reported as median (interquartile range, 25th–75th percentile). Categorical variables were analyzed using the Chi-square test, while comparisons between independent groups were performed with either one-way ANOVA or the Kruskal–Wallis test, selected according to variance homogeneity and data normality. As a multiple comparison test, the Tukey–Kramer test was used after one-way ANOVA, and the Dunn test was used after the Kruskal–Wallis test. Multiple comparison tests were performed at the α = 0.05 level. The direction and level of the relationship between numerical variables were determined by correlation analysis. Pearson correlation was used for groups showing a normal distribution, and Spearman correlation was used for groups that did not. A significance level of *p* < 0.05 was used in interpreting the results.

## 3. Results

Eighty participants were recruited for the research, including 40 individuals with lung cancer (87.5% male) and an equal number of healthy controls (85% male). The lung cancer group was classified according to histological type as follows: 15% small cell lung cancer, 42.5% squamous cell carcinoma, and 42.5% adenocarcinoma. Regarding disease stage, 12.5% of the patients were at stage I, 15% at stage II, 25% at stage III, and 47.5% at stage IV. Metastasis was absent in 52.5% of the patients, whereas 32.5% showed a positive molecular test result (Table 1).

The hematological and biochemical characteristics of the control and lung cancer groups are compared in Table 2. WBC and neutrophil counts were significantly higher in the lung cancer group compared to the controls (*p* < 0.0001 for both). Similarly, monocyte count and MPV were also elevated in the patient group (*p* < 0.0001 and *p* = 0.013, respectively). Hemoglobin levels were significantly lower in the lung cancer group (*p* = 0.006). No significant differences were found in lymphocyte and platelet counts between the two groups (*p* > 0.05). Regarding biochemical parameters, CRP, urea, AST, and LDH levels were significantly higher in the lung cancer group (*p* < 0.05 for all), whereas ALT levels were significantly lower compared to controls (*p* = 0.001) (Table 2).

These findings suggest increased systemic inflammation and altered liver enzyme activity in patients with lung cancer, which may be associated with enhanced OS and impaired antioxidant defense mechanisms reflecting the metabolic burden of the disease and tumor-related tissue damage. Systemic inflammation and OS are closely interconnected processes in lung cancer. Proinflammatory cells, particularly activated neutrophils and monocytes, are major sources of ROS through mechanisms such as respiratory burst and activation of NADPH oxidase. Elevated inflammatory markers, including CRP and leukocyte subtypes, may therefore directly contribute to increased systemic OS. In turn, excessive ROS can amplify inflammatory signaling by activating redox-sensitive pathways, leading to a self-perpetuating cycle of inflammation and oxidative imbalance. This bidirectional interaction may partly explain the concurrent elevation of inflammatory indices and OS markers observed in our cohort and reflects the metabolic and inflammatory burden imposed by tumor-related tissue damage.

Upon comparison of OS markers between the two groups, both TT and NT levels were markedly reduced in patients with lung cancer compared with controls (*p* < 0.0001 for both). In contrast, disulfide levels were markedly higher in the patient group (*p* = 0.008). Accordingly, both disulfide/NT ratio and disulfide/TT ratio were significantly increased in lung cancer patients (*p* < 0.0001 for both), while the NT/TT ratio was lower (*p* < 0.0001). Furthermore, TOS and OSI (*p* < 0.0001 for both) were significantly higher in lung cancer patients, while TAS showed a slight but significant elevation compared to controls (*p* = 0.004) (Table 3).

Correlation analysis revealed significant associations between OS markers and several hematological and biochemical parameters. Monocyte count showed a strong negative correlation with TT and NT levels (r = −0.490, *p* < 0.0001; r = −0.527, *p* < 0.0001, respectively) and a positive correlation with TOS and OSI (r = 0.696 and r = 0.576, both *p* < 0.0001). Similarly, urea and CRP levels were negatively correlated with thiol parameters but positively correlated with TOS and OSI (all *p* < 0.0001). Neutrophil count was also positively associated with TOS (r = 0.476, *p* < 0.0001) and OSI (r = 0.442, *p* < 0.0001). The strong correlations of monocyte, neutrophil, and CRP levels with OS markers suggest that inflammatory cell activity has a significant impact on oxidative balance. In particular, the decrease in antioxidant defense capacity (low thiol levels) and the increase in oxidative load (high TOS/OSI) can be interpreted as the biochemical reflection of systemic inflammation (Table 4).

Analysis revealed no statistically meaningful variation in OS parameters among adenocarcinoma, squamous cell carcinoma, and small cell lung cancer subtypes (*p* > 0.05 for all comparisons). Median TT levels were similar across subtypes, measuring 348 µmol/L in adenocarcinoma, 348 µmol/L in small cell carcinoma, and 325 µmol/L in squamous cell carcinoma. Likewise, NT concentrations and disulfide levels showed comparable values across groups. A modest elevation in the disulfide/NT and disulfide/TT ratios was observed in squamous cell carcinoma cases (median: 7.5% and 6.5%, respectively) compared with adenocarcinoma and small cell carcinoma; however, this variation failed to reach statistical significance (*p* = 0.053). TAS, TOS, and OSI values also showed no significant variation among histological subtypes, suggesting that oxidative balance is not markedly influenced by tumor histology (Table 5).

When OS parameters were compared across clinical stages of lung cancer, no statistically significant differences were observed among the groups (*p* > 0.05 for all). Although TT and NT levels tended to decrease and oxidative indices such as disulfide-related ratios, TOS, and OSI tended to increase with advancing stage, these variations were not significant. This suggests that the oxidative imbalance is present throughout disease progression rather than being restricted to a specific stage (Table 6).

Comparison of OS parameters according to metastasis status revealed that NT levels were significantly higher in patients with metastasis compared to those without (*p* = 0.022), while TT levels showed a similar but non-significant trend (*p* = 0.057). Other OS markers, including disulfide concentrations, thiol/disulfide ratios, TAS, TOS, and OSI, showed no significant differences between the groups (Table 7).

This pattern indicates that the antioxidant defense system, reflected by NT levels, might undergo a compensatory upregulation in patients with metastatic disease.

When OS parameters were compared according to molecular testing status, no statistically significant differences were found among mutation-positive, -negative, and untested groups (*p* > 0.05 for all). Nonetheless, both TT and NT levels tended to be higher in patients with positive molecular test results, while TOS and OSI values were slightly lower compared to the other groups. This trend may indicate that patients harboring targetable molecular alterations exhibit a relatively balanced oxidative status, possibly reflecting different tumor biology or host response profiles (Table 8).

To evaluate the diagnostic performance of OS parameters in lung cancer, an ROC analysis was performed. The optimal cutoff value for TT (µmol/L) was determined as 402.3, with a sensitivity of 88% and a specificity of 88%. For NT (µmol/L), the optimal cutoff value was 368.1, yielding a sensitivity of 98% and a specificity of 88%. The optimal cutoff value for disulfide was 15.5, with a sensitivity of 93% and a specificity of 43% (Table 9).

**Figure 1:**
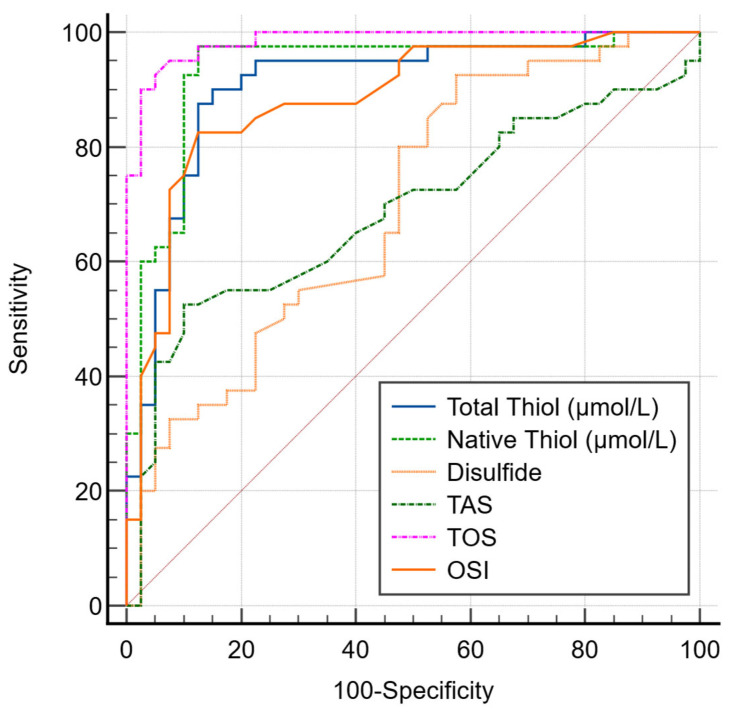
ROC curve comparison of OS markers for lung cancer.

The AUC values of OS parameters TT (µmol/L), NT (µmol/L), and disulfide were found to be 0.907, 0.934, and 0.694, respectively (Figure 1).

## 4. Discussion

Previous studies have demonstrated that NT and TT levels are significantly reduced in lung cancer patients, indicating a disruption of oxidant/antioxidant homeostasis and highlighting the involvement of OS in lung cancer pathogenesis [17,18,19]. Unlike these investigations, the present study provides a more comprehensive evaluation by simultaneously assessing TDH together with TAS, TOS, and OSI, thereby allowing an integrated assessment of both specific and global OS markers.

Our findings are consistent with previous reports that have demonstrated changes in thiol/disulfide parameters in patients with lung cancer. The study by Yalcin and colleagues evaluated TDH in 150 patients with lung cancer and reported significantly higher disulfide, thiol/disulfide (%), and disulphide/TT (%) values in patients with advanced stage disease compared with those at early stages, whereas NT and TT levels did not differ significantly between groups [20]. These findings suggest that oxidative imbalance becomes more pronounced with disease progression. In our study, although no significant differences were observed between stages, TT and NT levels were markedly reduced and disulfide levels were elevated in lung cancer patients compared to healthy controls, indicating a shift toward an oxidized state. Taken together, both studies emphasize that disruption of TDH reflects increased OS and may be associated with tumor aggressiveness and progression, even if stage-related variations are not always evident.

Similarly, a prospective controlled study including untreated lung cancer patients and healthy controls was conducted, which demonstrated significantly lower NT and TT levels in the patient group (*p* < 0.001), while disulfide levels did not differ significantly between the two groups [18]. These findings indicate that the reduction in thiol levels may reflect the depletion of antioxidant reserves in lung cancer. Our observations in this study were in agreement with these results, as both TT and NT concentrations were significantly lower and disulfide levels were elevated in lung cancer patients compared to controls, suggesting enhanced oxidative conversion of thiols. Although the previous study did not find a significant difference in disulfide levels, this discrepancy might be related to differences in patient characteristics, disease heterogeneity, or analytical sensitivity. Collectively, the findings of both studies indicate an oxidative imbalance in lung cancer and highlight TDH as a potential biochemical marker of redox disturbance.

In another study involving 35 patients with advanced non-small cell lung cancer (NSCLC) and 35 healthy controls, it was reported that NT, TT, and disulfide levels were all significantly lower in patients than in controls [17]. Moreover, reduced NT and disulfide concentrations were associated with poorer overall survival and lower performance status, suggesting that depletion of thiol components reflects both OS and disease aggressiveness. In contrast, our study found elevated disulfide levels along with decreased thiol concentrations in lung cancer patients compared with healthy individuals, indicating a shift toward a more oxidized redox state. This difference might be attributed to the inclusion of various histological subtypes and disease stages in our cohort, whereas the previous study focused exclusively on advanced-stage NSCLC. Nevertheless, both studies highlight that alterations in thiol/disulfide equilibrium are closely linked to tumor biology and may have prognostic implications in lung cancer.

In a prospective case–control design including 60 individuals with metastatic or inoperable NSCLC and an equal number of matched healthy participants, serum concentrations of TT, NT, and disulfide were found to be significantly decreased in patients relative to controls [21]. Furthermore, a low disulfide level was identified as an independent predictor of poor survival. These findings support the role of impaired TDH in the pathogenesis and prognosis of NSCLC. In contrast, our study demonstrated reduced TT and NT levels but elevated disulfide concentrations in the lung cancer group. This pattern may reflect a relative shift toward oxidative processes or adaptive redox responses in the tumor microenvironment; however, this interpretation remains hypothetical and cannot be directly confirmed by the present data. Elevated NT levels observed in certain subgroups, including metastatic patients, should therefore be interpreted cautiously, as they may arise from multiple, non-mutually exclusive mechanisms such as altered systemic redox buffering, tumor-related metabolic reprogramming, inflammatory activity, or host-related factors rather than a definitive compensatory antioxidant activation. However, while our study focused on the diagnostic performance of these markers using ROC analysis, Karataş emphasized their prognostic significance. This discrepancy might stem from differences in disease stage, tumor burden, or redox adaptation in distinct patient populations. Nevertheless, both studies underscore the involvement of OS and redox imbalance in lung cancer biology and highlight the potential prognostic relevance of thiol/disulfide parameters. Although ROC analyses in our cohort demonstrated high discriminatory performance for certain OS markers, these results should be interpreted with caution. The present study was conducted in a relatively small, single-center population and did not include either external validation or internal validation procedures such as bootstrapping. Therefore, the observed diagnostic performance should be regarded as exploratory rather than definitive. Rather than establishing diagnostic accuracy, our findings suggest a potential biological and clinical signal that warrants further investigation.

Broader investigations on OS parameters have yielded comparable findings. A study examined OS parameters, including TAS, TOS, and OSI, in 94 lung cancer patients and 64 healthy controls [22]. In contrast to this study, our findings demonstrated preserved or relatively increased TAS levels despite clear evidence of oxidative imbalance, suggesting that systemic antioxidant capacity may not necessarily reflect effective redox control in lung cancer patients. Although oxidative markers tended to worsen with advancing cancer stage, these differences did not reach statistical significance. These results are consistent with our observation of elevated disulfide levels and reduced thiol concentrations, both of which indicate a shift toward oxidation and impairment of redox homeostasis. Notably, TAS represents the cumulative activity of circulating antioxidant components rather than effective intracellular antioxidant capacity. Therefore, in the context of chronic OS and systemic inflammation, variations in TAS may reflect a compensatory or adaptive systemic response to increased oxidant burden rather than preserved antioxidant defense. Taken together, these findings reinforce the concept that systemic oxidative imbalance plays a central role in the development and progression of lung cancer, regardless of the specific OS biomarker system employed. One of the notable findings of the present study was the higher TAS levels observed in patients with lung cancer, which may appear contradictory to the concept of impaired antioxidant defense in malignancy. However, TAS represents the cumulative contribution of multiple circulating antioxidants, including non-enzymatic components such as uric acid, bilirubin, and albumin, rather than a direct measure of effective antioxidant protection [23]. In the context of chronic OS and systemic inflammation, elevated TAS may reflect a compensatory or reactive upregulation of antioxidant components in response to increased oxidant load. Moreover, inflammatory and metabolic alterations frequently observed in lung cancer may influence TAS values independently of true redox balance. The lack of stratified analyses based on nutritional status or specific antioxidant contributors (e.g., albumin or uric acid) constitutes a limitation of this study and should be addressed in future investigations to better elucidate the biological significance of elevated TAS in lung cancer.

In addition, a study demonstrated that total antioxidant capacity (TAC) in lung cancer patients was positively correlated with serum albumin and uric acid levels, and inversely correlated with CRP, suggesting that endogenous antioxidant mechanisms are closely linked with systemic inflammation and nutritional status [24]. Notably, TAC was significantly associated with disease stage, whereas lifestyle factors such as diet and smoking showed no significant influence. These findings imply that redox homeostasis in lung cancer is primarily determined by tumor-related metabolic alterations rather than external factors. Similarly, in our study, changes in TDH, characterized by reduced thiol levels and increased disulfide formation, reflect intrinsic OS associated with the disease process itself. Together, these data support the notion that oxidative imbalance is an inherent feature of lung cancer pathophysiology rather than a consequence of modifiable lifestyle factors.

A recent review further summarized the growing evidence linking systemic OS to cancer development, emphasizing that serum thiols are robust and sensitive indicators of redox status [25]. The review highlighted consistent reductions in TT and NT levels, as well as alterations in disulfide concentrations, across various cancer types including lung cancer. Importantly, lower thiol levels were associated with decreased overall survival, reinforcing the prognostic relevance of TDH. Our results align with this comprehensive view, suggesting that evaluation of thiol/disulfide balance may serve as a practical and reproducible biomarker reflecting the oxidative milieu in lung cancer patients.

This study has several limitations. The relatively small, single-center cohort limits generalizability. Although ROC analyses suggested good discriminatory performance for some OS markers, the lack of external or internal validation means that these results should be considered exploratory rather than definitive.

All analyses were univariate, and multivariable models adjusting for inflammatory and metabolic confounders such as CRP, leukocyte subtypes, LDH, and renal function parameters were not performed; therefore, independent associations could not be established. Subgroup analyses by histology, stage, metastatic status, and molecular characteristics were limited by very small sample sizes and should be interpreted cautiously.

The cross-sectional design precludes causal or prognostic inferences, and alterations in TDH may reflect adaptive redox responses rather than direct pathogenic mechanisms. Future multicenter, longitudinal studies with larger cohorts and validated models are warranted.

## 5. Conclusions

In conclusion, our findings correlate a significant disruption of TDH and increased OS in patients with lung cancer compared to healthy individuals. The observed decrease in TT and NT levels, increased disulfide formation, and elevated oxidative indices indicate a shift toward oxidative imbalance, which may contribute to tumor pathogenesis and progression. Although no significant differences were observed among histological subtypes or disease stages, the consistent presence of oxidative alterations suggests that redox dysregulation is a fundamental feature of lung cancer biology. These results highlight the potential utility of assessing TDH as a simple and reproducible biomarker for evaluating oxidative status in lung cancer patients. Future longitudinal and mechanistic studies are warranted to explore the clinical relevance of these redox parameters and to determine whether restoring oxidative balance could offer novel therapeutic opportunities in lung cancer treatment.

## Figures and Tables

**Table 1:** Demographic features of the groups.

		Groups				*p*-Value
		Control		Lung Cancer		
		n = 40		n = 40		
		Mean	SD	Mean	SD	
Age (years)		67.2	6.7	66.9	8.6	0.897
		**n**	**%**	**n**	**%**	
Gender	Female	6	15.0	5	12.5	0.747
	Male	34	85.0	35	87.5	
Histological type	Adenocarcinoma			17	42.5	
	Small cell lung cancer			6	15	
	Squamous cell carcinoma			17	42.5	
Stage	1			5	12.5	
	2			6	15	
	3			10	25	
	4			19	47.5	
Metastasis	No			21	52.5	
	Yes			19	47.5	
Molecular testing	Negative			4	10	
	Not examined			23	57.5	
	Positive			13	32.5	

**Table 2:** Comparison results on laboratory parameters in the control and lung cancer groups.

	Groups				*p*-Value
	Control		Lung Cancer		
	n = 40		n = 40		
Hematological results					
WBC (10^3^/L)	7.2	2.3	9.9	2.9	<0.0001 *
Neutrophil(10^3^/L)	3.5	(2.8–4.9)	6.5	(4.9–8.5)	<0.0001 **
Lymphocyte(10^3^/L)	1.9	1.1	2.1	0.9	0.265
Monocyte (10^3^/L)	0.3	(0.2–0.5)	0.7	(0.6–1)	<0.0001 **
Platelet (10^3^/L)	260	(165–332)	297	(235–359)	0.070
MPV (fL)	9.4	(8.3–9.6)	9.7	(9–10.4)	0.013 **
Hemoglobin (g/dL)	13.5	(12.6–15.2)	13.0	(11.5–13.9)	0.006 **
Biochemical results					
CRP (mg/L)	3.0	(2–4)	10.0	(4.9–19.5)	<0.0001 **
Urea (mg/dL)	14.0	(12–17)	30.5	(23–38)	<0.0001 **
ALT (IU/L)	25.5	(18.5–30)	17.0	(13–22)	0.001 **
AST (IU/L)	17.5	6.0	22.3	10.9	0.016 *
LDH (IU/L)	167	(148–189)	224	(186–255)	<0.0001 **

* Significant difference at <0.05 level according independent *t*-test, Means and Standard deviations (SD) are presented. ** Significant difference at <0.05 level according to Mann–Whitney U test, Medians are presented and 25p–75p are shown in parentheses.

**Table 3:** Comparison of OS parameters between patients and control group.

OS Markers	Groups				*p*-Value
	Control		Lung Cancer		
	n = 40		n = 40		
TT (µmol/L)	437.0	(417–462)	346.2	(320–395)	<0.0001 **
NT (µmol/L)	398.0	(387–419)	297.8	(280–333)	<0.0001 **
Disulfide	18.6	8.1	23.3	7.5	0.008 *
Disulfide/NT (%)	4.54	(3.53–5.22)	7.11	(5.7–9.7)	<0.0001 **
Disulfide/TT (%)	4.16	(3.29–4.72)	6.23	(5.1–8.2)	<0.0001 **
NT/TT (%)	91.7	(90.6–93.4)	87.5	(84–90)	<0.0001 **
TAS	1.16	(1.03–1.26)	1.35	(1.1–1.5)	0.004 **
TOS	3.19	0.67	5.62	0.95	<0.0001 *
OSI	0.28	0.09	0.44	0.13	<0.0001 *

* Significant difference at <0.05 level according independent *t*-test, Means and Standard deviations (SD) are presented. ** Significant difference at <0.05 level according to Mann–Whitney U test, Medians are presented and 25p–75p are shown in parentheses.

**Table 4:** Correlation analysis findings between laboratory parameters and OS markers.

		TT (µmol/L)	NT (µmol/L)	Disulfide	TAS	TOS	OSI
		n = 80					
WBC (10^3^/L)	*r*	−0.203	−0.220	0.173	0.125	0.399	0.363
	*p*-Value	0.071	0.050	0.125	0.271	0.000 *	0.001 *
Neutrophil (10^3^/L)	*r*	−0.423	−0.421	0.176	0.107	0.476	0.442
	*p*-Value	0.000 **	0.000 **	0.119	0.344	<0.0001 **	<0.0001 **
Lymphocyte(10^3^/L)	*r*	0.014	−0.018	0.137	0.093	0.147	0.138
	*p*-Value	0.901	0.874	0.225	0.413	0.193	0.222
Monocyte (10^3^/L)	*r*	−0.490	−0.527	0.311	0.257	0.696	0.576
	*p*-Value	<0.0001 **	<0.0001 **	0.005 **	0.021 **	<0.0001 **	<0.0001 **
Platelet (10^3^/L)	*r*	−0.097	−0.158	0.217	0.143	0.247	0.168
	*p*-Value	0.391	0.162	0.053	0.206	0.027**	0.138
MPV (fL)	*r*	−0.240	−0.213	−0.101	0.126	0.182	0.077
	*p*-Value	0.032 **	0.058	0.374	0.267	0.106	0.497
Hemoglobin (g/dL)	*r*	0.179	0.212	−0.234	−0.266	−0.221	−0.099
	*p*-Value	0.112	0.059	0.037 **	0.017 **	0.049 **	0.381
CRP (mg/L)	*r*	−0.426	−0.498	0.303	0.258	0.530	0.404
	*p*-Value	0.000 **	<0.0001 **	0.006 **	0.021 **	<0.0001 **	0.000 **
Urea (mg/dL)	*r*	−0.557	−0.589	0.330	0.171	0.684	0.582
	*p*-Value	<0.0001 **	<0.0001 **	0.003 **	0.129	<0.0001 **	<0.0001 **
ALT (IU/L)	*r*	0.039	0.099	−0.280	−0.076	−0.263	−0.239
	*p*-Value	0.732	0.381	0.012 **	0.500	0.018 **	0.033 **
AST (IU/L)	*r*	−0.253	−0.210	−0.028	0.087	0.283	0.150
	*p*-Value	0.024 **	0.062	0.807	0.442	0.011 *	0.183
LDH (IU/L)	*r*	−0.398	−0.413	0.134	0.227	0.521	0.446
	*p*-Value	0.000 **	0.000 **	0.235	0.043 **	<0.0001 **	<0.0001 **

* Significant correlation coefficient at <0.05 level according to Pearson correlation. ** Significant correlation coefficient at <0.05 level according Spearman rank correlation.

**Table 5:** Comparative analysis of OS markers in histological types.

	Histological Type									*p*-Value
	Adenocarcinoma			Small Cell			Squamous Cell			
	n = 17			n = 6			n = 17			
	Median	25p	75p	Median	25p	75p	Median	25p	75p	
TT (µmol/L)	348	330	396	348	331	374	325	308	367	0.277
NT (µmol/L)	302	286	354	307	295	338	286	259	315	0.146
Disulfide	21.2	16.3	27.3	18.5	18.2	19.4	23.3	19.4	32.6	0.146
Disulfide/NT (%)	6.5	4.9	9.6	5.8	5.2	6.9	7.5	7.0	11.4	0.053
Disulfide/TT (%)	5.8	4.5	8.0	5.2	4.8	6.1	6.5	6.1	9.3	0.053
NT/TT (%)	88.5	83.9	91.1	89.6	87.9	90.5	87.0	81.5	87.8	0.054
TAS	1.3	1.2	1.8	1.4	1.1	1.5	1.4	1.1	1.4	0.722
TOS	5.4	4.9	5.9	5.9	5.3	6.3	6.1	4.5	6.5	0.719
OSI	0.41	0.32	0.49	0.41	0.40	0.55	0.45	0.38	0.51	0.579

**Table 6:** Comparison of OS markers across stages.

	Stages												*p*-Value
	Stage 1			Stage 2			Stage 3			Stage 4			
	n = 5			n = 6			n = 10			n = 19			
	Median	25p	75p	Median	25p	75p	Median	25p	75p	Median	25p	75p	
TT (µmol/L)	348	326	395	320	309	355	342	314	348	350	327	401	0.332
NT (µmol/L)	294	288	332	274	259	286	296	279	312	308	289	356	0.076
Disulfide	20.2	18.3	30.0	31.1	18.7	34.2	21.1	18.2	26.3	21.2	17.8	25.0	0.612
Disulfide/NT (%)	6.5	5.7	10.0	10.7	7.2	12.7	7.1	6.1	8.9	7.0	5.1	8.6	0.320
Disulfide/TT (%)	5.8	5.1	8.3	8.8	6.3	10.1	6.2	5.4	7.6	6.2	4.6	7.4	0.320
NT/TT (%)	88.5	83.4	89.8	82.4	79.8	87.4	87.6	84.9	89.2	87.7	85.3	90.8	0.320
TAS	1.6	1.0	2.0	1.3	1.1	1.5	1.4	1.2	1.5	1.3	1.2	1.5	0.787
TOS	5.3	4.8	5.7	4.9	4.2	6.5	6.2	6.1	6.6	5.6	4.8	6.1	0.139
OSI	0.41	0.26	0.50	0.42	0.38	0.46	0.42	0.39	0.57	0.45	0.36	0.50	0.734

**Table 7:** Comparison results on OS markers in the metastasis status.

	Metastasis Status				*p*-Value
	Not Present		Present		
	n = 21		n = 19		
	Mean	SD	Mean	SD	
TT (µmol/L)	341	32	366	48	0.057
NT (µmol/L)	292	31	321	44	0.022 *
Disulfide	24.2	7.6	22.4	7.5	0.444
Disulfide/NT (%)	8.4	3.0	7.1	2.4	0.121
Disulfide/TT (%)	7.1	2.2	6.1	1.8	0.125
NT/TT (%)	85.8	4.3	87.8	3.6	0.125
TAS	1.4	0.4	1.3	0.3	0.528
TOS	5.7	1.1	5.5	0.8	0.397
OSI	0.44	0.15	0.44	0.11	0.822

* Significant difference at <0.05 level according independent *t*-test, Means and Standard deviations (SD) are presented.

**Table 8:** Comparison results on OS markers in the molecular testing.

	Molecular Testing									*p*-Value
	Negative			Not Examined			Positive			
	n = 4			n = 23			n = 13			
	Median	25p	75p	Median	25p	75p	Median	25p	75p	
TT (µmol/L)	322	309	334	347	314	370	349	330	399	0.088
NT (µmol/L)	287	262	296	294	278	322	316	295	355	0.093
Disulfide	19.0	17.7	24.6	22.6	18.6	31.0	22.0	16.3	27.1	0.601
Disulfide/NT (%)	6.4	6.2	9.3	7.2	5.9	9.8	7.1	4.8	9.6	0.437
Disulfide/TT (%)	5.7	5.5	7.7	6.3	5.3	8.2	6.2	4.4	8.0	0.437
NT/TT (%)	88.7	84.5	89.0	87.4	83.6	89.5	87.6	83.9	91.2	0.437
TAS	1.4	1.0	1.7	1.4	1.1	1.6	1.3	1.2	1.4	0.834
TOS	6.7	5.9	7.5	5.4	5.0	6.4	5.7	4.8	5.9	0.138
OSI	0.48	0.38	0.77	0.41	0.38	0.49	0.44	0.35	0.49	0.731

**Table 9:** Diagnostic efficacy of OS indicators in identifying lung cancer.

	Cut-Off	Sensitivity	Specificity	PPV	NPV	AUC (95% CI)		*p*-Value
TT (µmol/L)	≤402.3	88	88	87.5	87.5	0.907	(0.821–0.960)	<0.0001
NT (µmol/L)	≤368.1	98	88	88.6	97.2	0.934	(0.856–0.978)	<0.0001
Disulfide	>15.5	93	43	61.7	85.0	0.694	(0.581–0.792)	0.001
TAS	>1.33	53	90	84.0	65.5	0.687	(0.573–0.786)	0.003
TOS	>4.25	95	93	92.7	94.9	0.985	(0.928–0.999)	<0.0001
OSI	>0.35	83	88	86.8	83.3	0.883	(0.792–0.944)	<0.0001

AUC: Area under curve; CI: Confidence interval.

## Data Availability

The original contributions presented in this study are included in the article. Further inquiries can be directed to the corresponding author.

## Abbreviations

The following abbreviations are used in this manuscript:

OS	Oxidative stress
TDH	Thiol/disulfide homeostasis
TAS	Total antioxidant status
TOS	Total oxidant status
OSI	Oxidative stress index
ROS	Reactive oxygen species
NT	Native thiol
TT	Total thiol
DTNB	2,2-dithiobisnitrobenzoic acid
CRP	C-reactive protein
ALT	Alanine aminotransferase
AST	Aspartate aminotransferase
LDH	Lactate dehydrogenase
WBC	White blood cell
MPV	Mean platelet volume
SD	Standard deviation
NSCLC	Non-small cell lung cancer
TAC	Total antioxidant capacity
